# Supplementary material for: Super-resolution imaging of native fluorescent photoreceptors in chytrid fungal eyes
Source: EMBO J. 2025 May 27;44(14):4088–103. doi: 10.1038/s44318-025-00452-x (PMC12264004; doi:10.1038/s44318-025-00452-x)
Supplement: Supplementary file 1 — Appendix [file 44318_2025_452_MOESM1_ESM.pdf]

Appendix

Super-Resolution Imaging of Native Fluorescent Photoreceptors in Chytrid Fungal Eyes

Wayne Busse, Enrico Klotzsch, Yousef Kamrani, Natalie Wordtmann, Simon Kelterborn, Peter Hegemann, Matthias Broser*

*Correspondence should be addressed to M. B. (matthias.broser@hu-berlin.de)

Table of contents

Appendix Table S1 2
Appendix Table S2 3
Appendix Table S3 4
Appendix Figure S1 5
References 6

Name	Ex	Em	Ext. Coeff.	QF	Bright.	CF
MRhubarb720	701	720	95000	65	6.13	BV
miRFP720	702	720	98000	61	5.98	BV
miRFP718nano	690	718	79000	56	4.42	BV
miRFP713	690	713	99000	0.07	6.93	BV
miRFP	674	703	92400	97	8.96	BV
miRFP680	661	680	94000	145	13.63	BV
miRFP670nano3	645	670	129000	185	23.86	BV
mCarmine	603	675	83000	0.07	5.81	*
mCardinal	604	659	87000	0.19	16.53	*
mNeptune2	599	651	89000	0.24	21.36	*
mKate2	588	633	62500	0.4	25	*
plobRFP	578	614	84000	0.74	62.16	*
GFP(S65T)	490	510	55000	0.64	35.2	*
*Cp*NeoR	720	735	169000	0.15	25.35	ATR
*Rg*NeoR	690	707	129000	0.2	25.8	ATR
GpNeoRh	630	668	100000	0.1	10	ATR
Archon2 (GEVI)	586	735	38800	11	0.41	ATR

Appendix Table S1. Properties of various fluorescent proteins, related to introduction. Ex; Em: wavelengths for maximal excitation and emission (nm); Ext. Coeff.: molar decadic extinction coefficient ($M^{-1}cm^{-1}$) at maximal excitation; QF: Fluorescence quantum yield; Brightness (10^4 x $M^{-1}cm^{-1}$); Cofactors (CF): Billiverdin (BV); *GFP variant; all-*trans* retinal (ATR). Color-code according to protein class: Phytochrome-based (red); GFP-based (blue) and rhodopsins (green). Data from FPbase (Lambert, 2019; Penzkofer et al, 2019; Broser et al, 2020; Broser et al, 2023; Oliinyk et al, 2023).

Name	#	Sequence
JEL800_NeoR_fwd	1	GGAAACACTTACATCCTCGTTGGA
JEL800_NeoR_Intron_fwd	2	GTGCTGCAGCATAATGCTTTTGA
JEL800_NeoR_rev	3	CGTTGAGACAACGGAGTGAAGT
JEL800_NeoR_full_fwd	4	ATGTCTGCACTCACCAAAGCA
JEL800_RGC1_full_fwd	5	ATGGACGACGTTCAGCGTG
JEL800_RGC1_fwd	6	TGCCACTCTGGAGGAGTCTATG
JEL800_RGC1_Intron_fwd	7	CAACATTCTTTCCATTTTCGCTTAGCT
JEL800_RGC1_rev	8	TCCGTTAGCTTTTCCTGCTTTCA
JEL800_RGC2_full_fwd	9	ATGACTGACCTTCAACGTGGG
JEL800_RGC2_fwd	10	ATGAGATTGAAAGCCAAATGGCTCA
JEL800_RGC2_Intron_fwd	11	GTAAGGGTCATATTCAACAAAGATATTGTTACTC
JEL800_RGC2_rev	12	GCAGCTCTCTCAGCATGGTC

Appendix Table S2. Primer used for RT-PCR on extracted RNA from *R. globosum* zoospores, related to Figure 2. JEL800 genome for primer design can be found on mycocosm.jgi.doe.gov/Rhihy1/ or source data.

		NeoR	RGC1	RGC2	#1 CNG-Protein (ID:719173)	#2 CNG-Protein (ID:718890)
zoospore	1	441.3463908	445.641569	181.6911077	0	14.29388645
	2	419.1253834	351.7181309	114.5327825	30.67807948	19.21460164
	3	343.3233448	177.9477092	87.95493629	33.12670408	7.407761151
germling	1	59.25029554	55.5204252	43.7080971	34.88103478	7.503433481
	2	59.27757547	69.61115531	32.14144422	31.82085772	4.172161552
	3	44.34927798	65.63656855	21.89874016	42.43613018	2.409066374
immature thallus	1	20.92856909	19.20386791	28.41999077	46.54023684	39.40895111
	2	20.81435042	20.86392428	27.17067172	43.08721737	25.95366301
	3	8.319956955	15.55244237	32.0978556	40.09582756	33.23559172
mixed	1	717.8180546	678.5447467	400.8181271	236.4659115	252.6429545
	2	495.4202948	368.1275826	223.7936039	151.3362602	147.7280771
	3	354.2767054	270.5765767	114.5597613	127.0768687	111.0597919

Appendix Table S3. Normalized RNA counts, related to Figure 3. RNA-count values for each stage in the *R. globosum* life cycle.

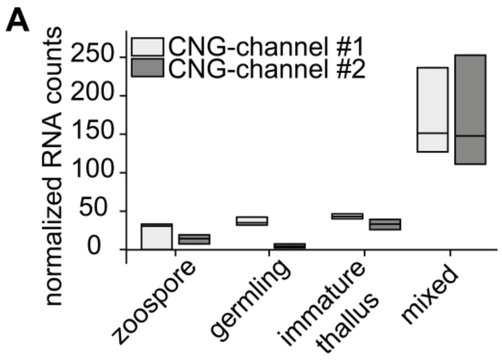

Appendix Figure S1. Transcriptome analysis of putative cGMP-sensitive ion channels, related to Figure 3. (A) Box-Plot of normalized RNA-sequencing counts for two putative cGMP-sensitive ion channels across the *R. globosum* lifecycle (Galindo et al, 2022), based on transcriptome analysis data published, n = 3 (biological replicates) (Laundon et al, 2022). Box plot displays the minimum, median (centre line), and maximum values.

References

- Broser, M., Spreen, A., Konold, P. E., Peter, E., Adam, S., Borin, V., Schapiro, I., Seifert, R., Kennis, J. T. M., Bernal Sierra, Y. A., & Hegemann, P. (2020). NeoR, a near-infrared absorbing rhodopsin. Nature Communications, 11(1), 2–11. 10.1038/s41467-020-19375-8.
- Broser, M., Busse, W., Spreen, A., Reh, M., Sierra, Y. A. B., Hwang, S., Utesch, T., Sun, H., & Hegemann, P. (2023). Diversity of rhodopsin cyclases in zoospore-forming fungi. Proc. Natl. Acad. Sci. USA, 120(44).10.1073/pnas.2310600120.
- Galindo, L. J., Milner, D. S., Gomes, S. L., & Richards, T. A. (2022). A light-sensing system in the common ancestor of the fungi. Current Biology, 1–8. 10.1016/j.cub.2022.05.034.
- Lambert, T. J. (2019). Fpbase: A Community-Editable Fluorescent Protein Database. Nat. Methods, 16 (4), 277-278. 10.1038/s41592-019-0352-8.
- Laundon, D., Chrismas, N., Bird, K., Thomas, S., Mock, T., & Cunliffe, M. (2022). A cellular and molecular atlas reveals the basis of chytrid development. ELife, 11. 10.7554/eLife.73933.
- Oliinyk, O. S., Ma, C., Pletnev, S., Baloban, M., Taboada, C., Sheng, H., Yao, J., Verkhusha, V. V. (2023). Deep-Tissue Swir Imaging Using Rationally Designed Small Red-Shifted near-Infrared Fluorescent Protein. Nat. Methods, 20 (1), 70-74. 10.1038/s41592-022-01683-0.
- Penzkofer, A.; Silapetere, A.; Hegemann, P. (2019). Absorption and Emission Spectroscopic Investigation of the Thermal Dynamics of the Archaerhodopsin 3 Based Fluorescent Voltage Sensor Quasar1. Int. J. Mol. Sci., 20 (17), 4086. 10.3390/ijms20174086.
