# Supplementary figures and images for: Super-resolution imaging of native fluorescent photoreceptors in chytrid fungal eyes
Source: EMBO J. 2025 May 27;44(14):4088–103. doi: 10.1038/s44318-025-00452-x (PMC12264004; doi:10.1038/s44318-025-00452-x)

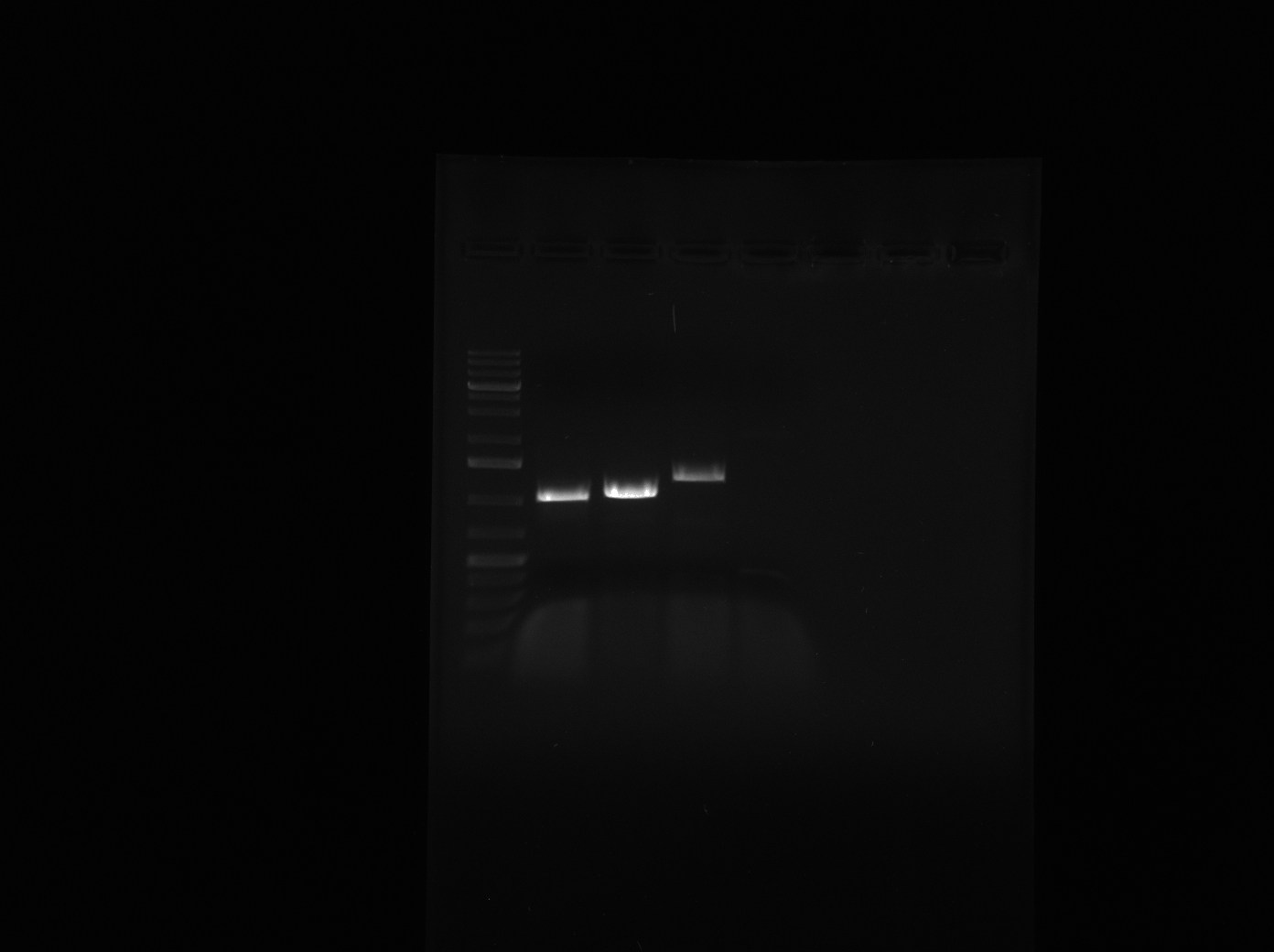

Supplement: Supplementary file 4 — Source data Fig. 2 [file 44318_2025_452_MOESM4_ESM.zip › 2B/DNA-gel_large.tif]

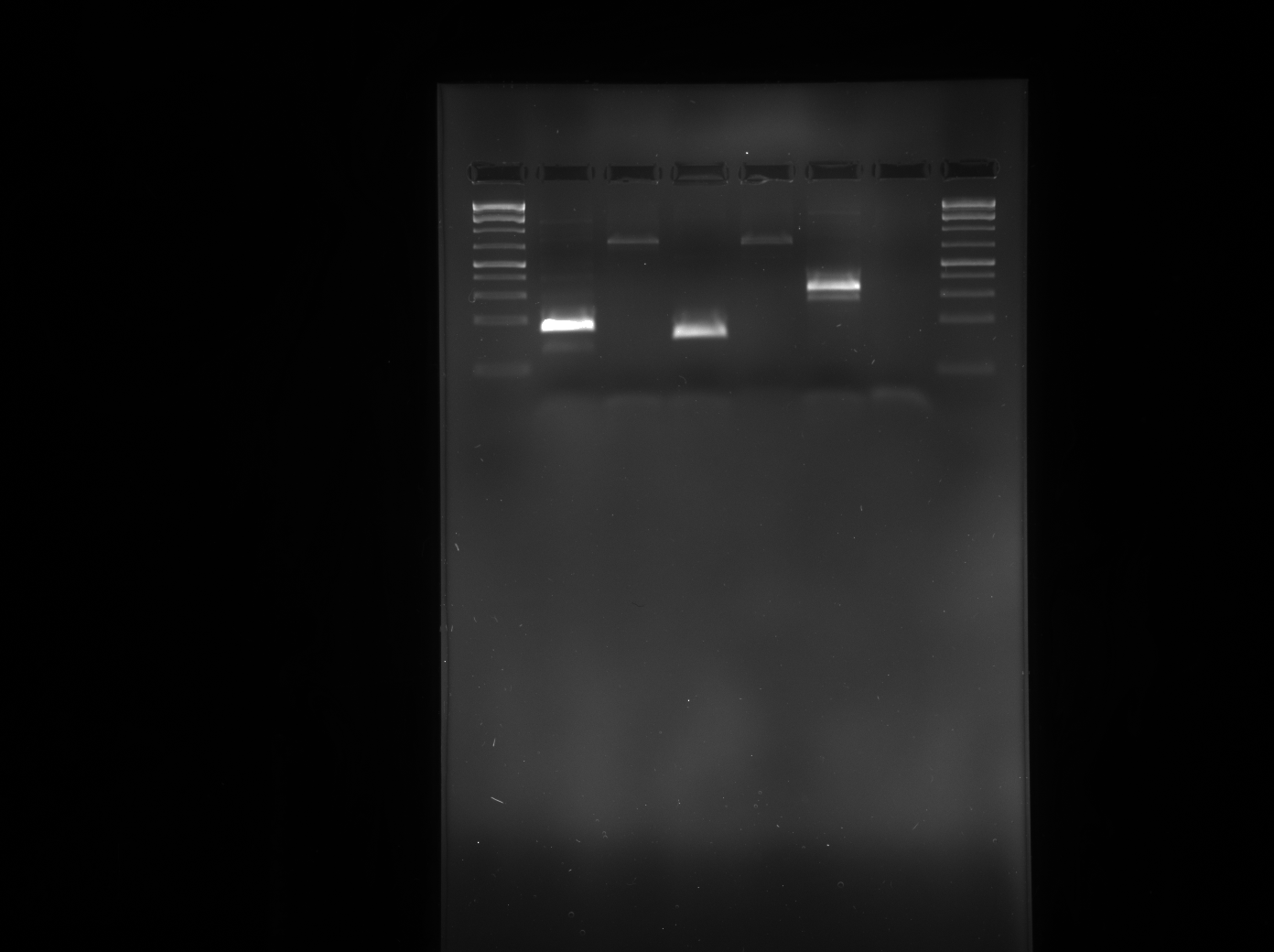

Supplement: Supplementary file 4 — Source data Fig. 2 [file 44318_2025_452_MOESM4_ESM.zip › 2B/DNA-gel_small.tif]

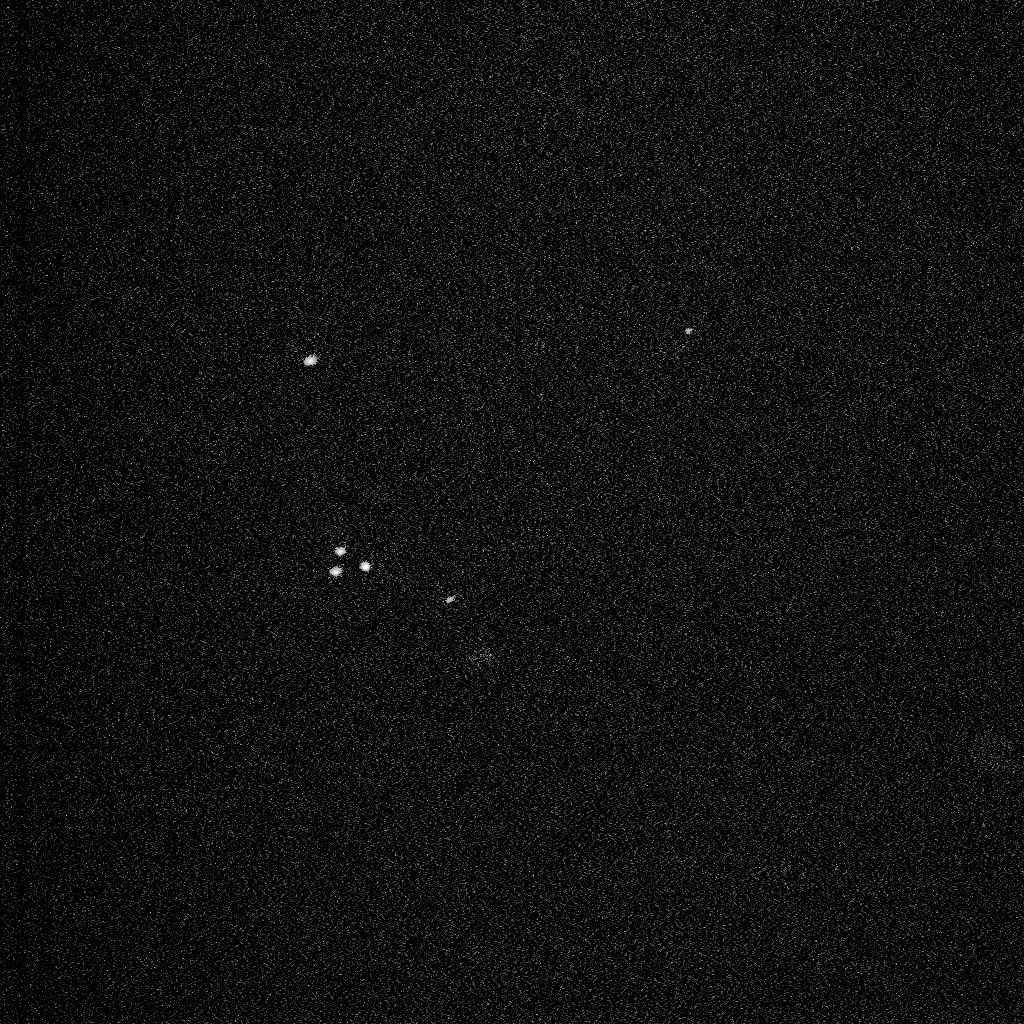

Supplement: Supplementary file 5 — Source data Fig. 3 [file 44318_2025_452_MOESM5_ESM.zip › 3B/exp2/RGZ3_bleach.tif]

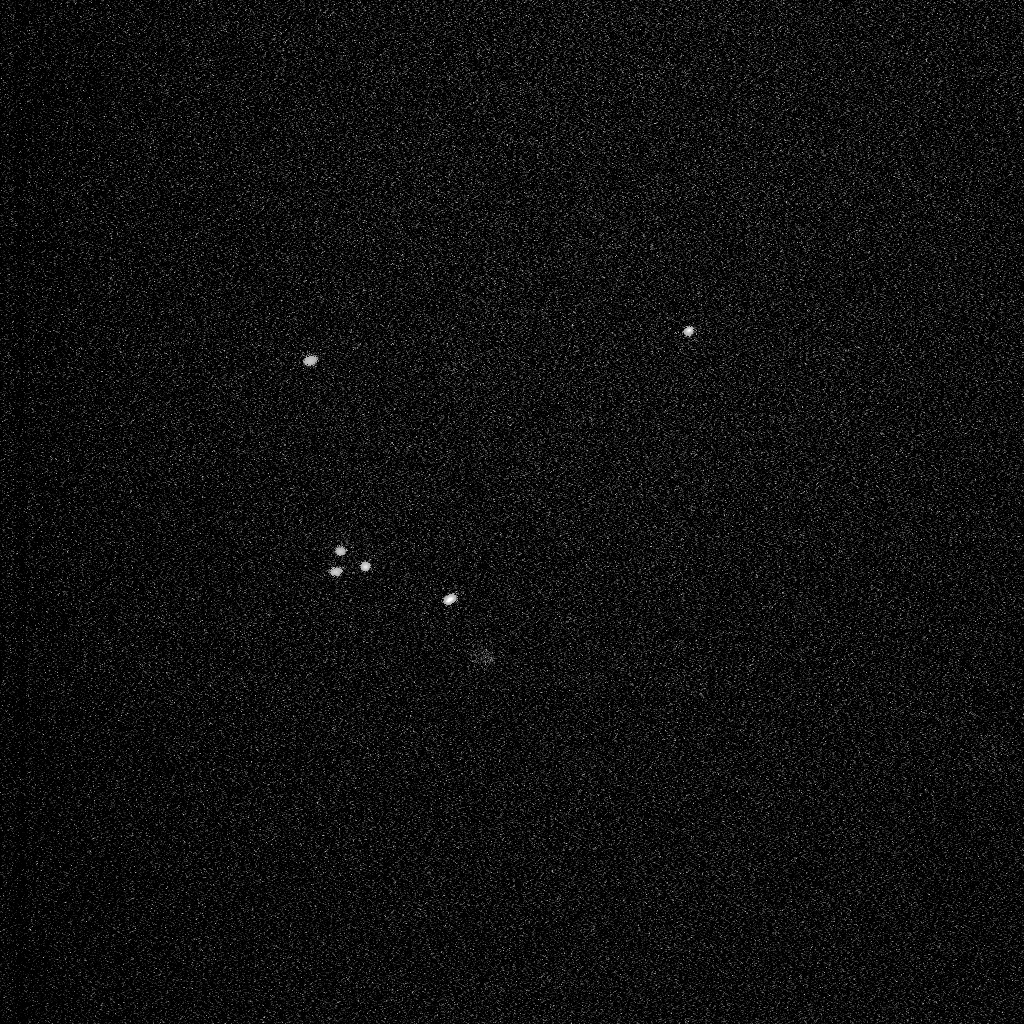

Supplement: Supplementary file 5 — Source data Fig. 3 [file 44318_2025_452_MOESM5_ESM.zip › 3B/exp2/RGZ3_initial.tif]

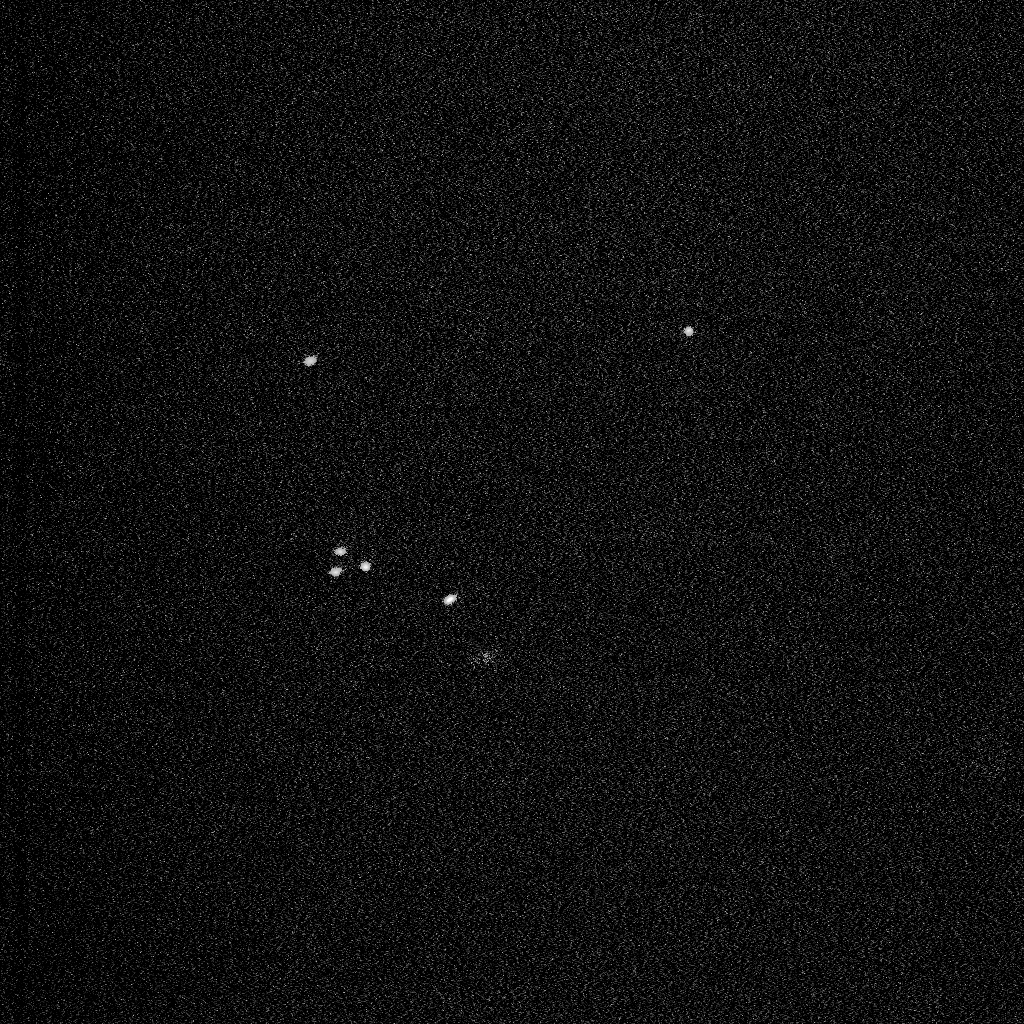

Supplement: Supplementary file 5 — Source data Fig. 3 [file 44318_2025_452_MOESM5_ESM.zip › 3B/exp2/RGZ3_recovery.tif]

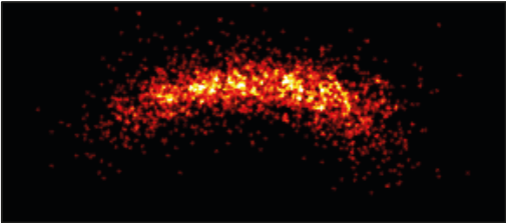

Supplement: Supplementary file 5 — Source data Fig. 3 [file 44318_2025_452_MOESM5_ESM.zip › 3C/Fig3C.pdf]

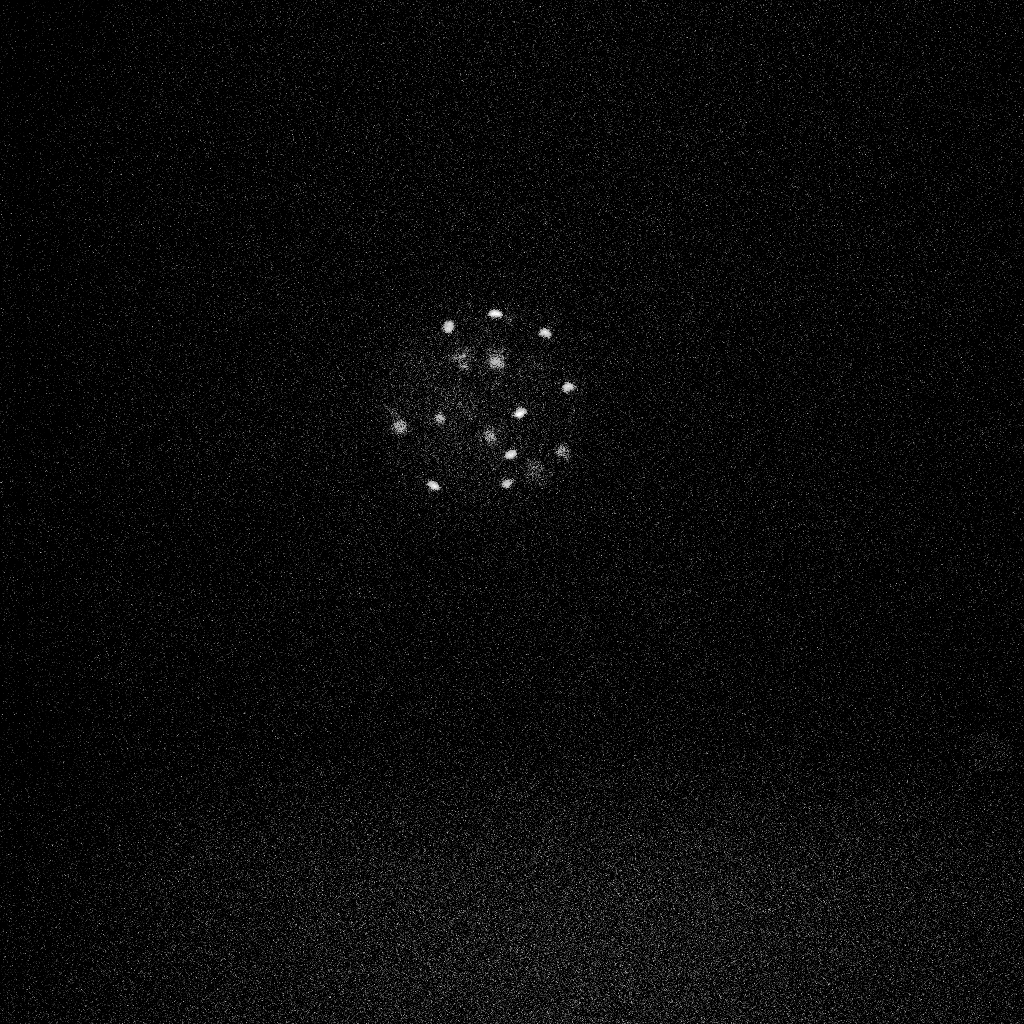

Supplement: Supplementary file 5 — Source data Fig. 3 [file 44318_2025_452_MOESM5_ESM.zip › 3E/z-stack_sporangium_640.tif]

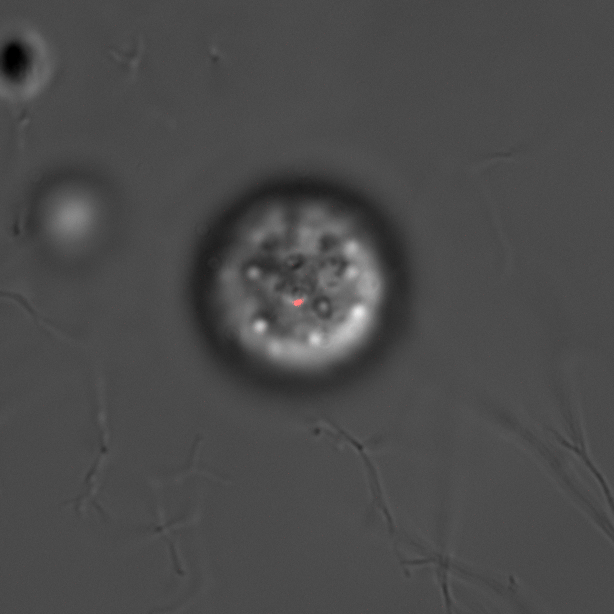

Supplement: Supplementary file 5 — Source data Fig. 3 [file 44318_2025_452_MOESM5_ESM.zip › 3E/Zstack0.png]

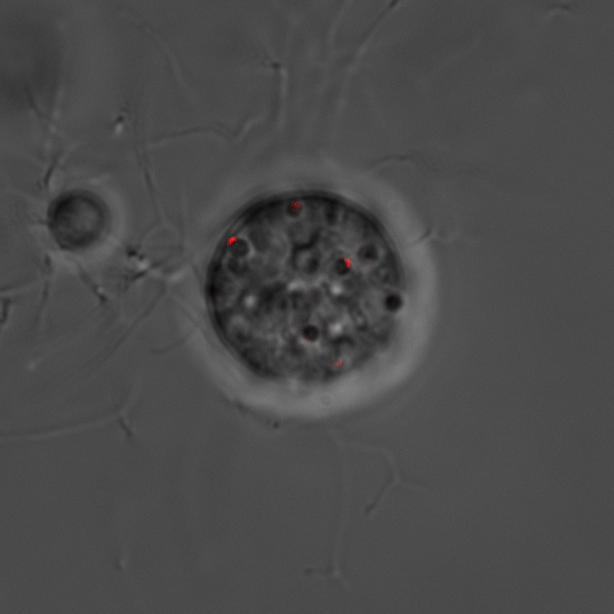

Supplement: Supplementary file 5 — Source data Fig. 3 [file 44318_2025_452_MOESM5_ESM.zip › 3E/Zstack105.png]

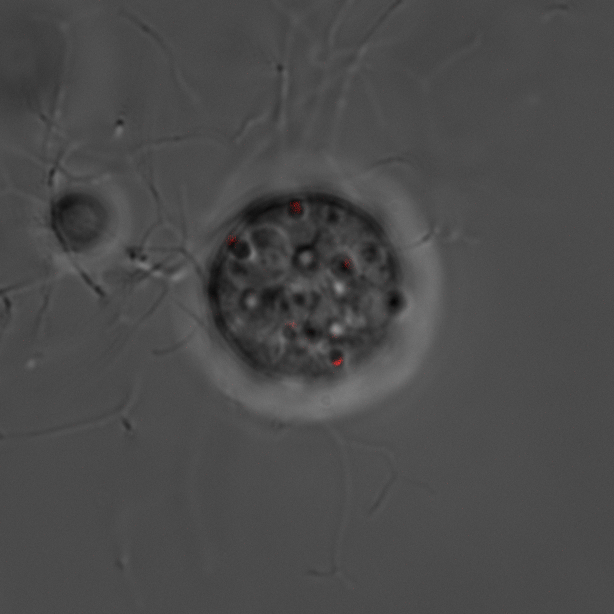

Supplement: Supplementary file 5 — Source data Fig. 3 [file 44318_2025_452_MOESM5_ESM.zip › 3E/Zstack112.png]

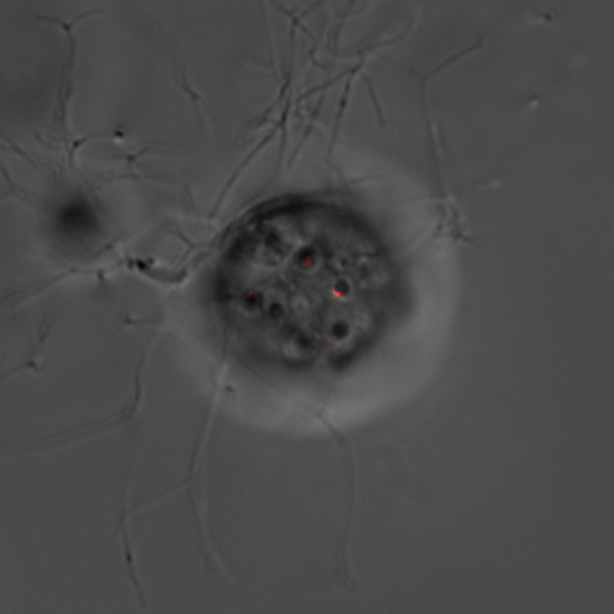

Supplement: Supplementary file 5 — Source data Fig. 3 [file 44318_2025_452_MOESM5_ESM.zip › 3E/Zstack140.png]

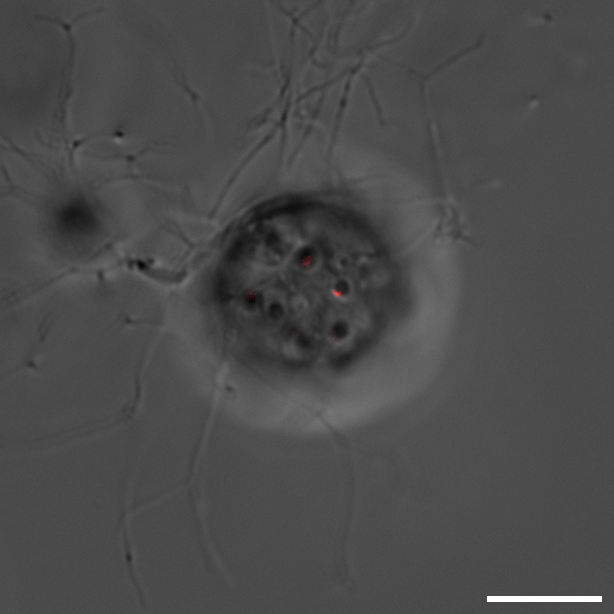

Supplement: Supplementary file 5 — Source data Fig. 3 [file 44318_2025_452_MOESM5_ESM.zip › 3E/Zstack140SB.png]

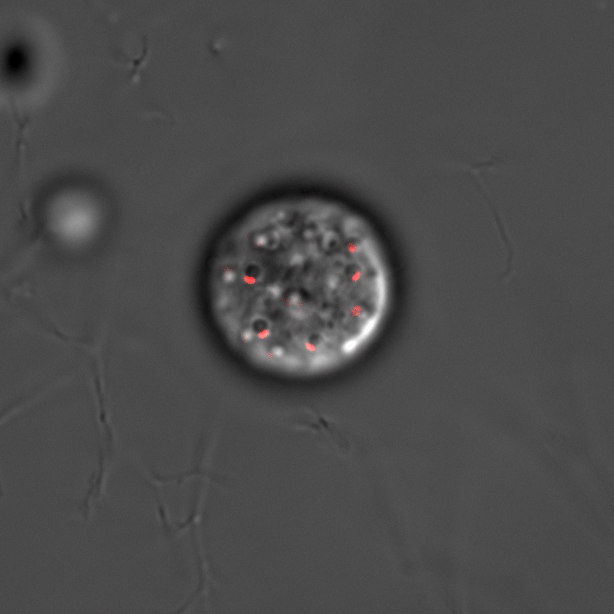

Supplement: Supplementary file 5 — Source data Fig. 3 [file 44318_2025_452_MOESM5_ESM.zip › 3E/Zstack21.png]

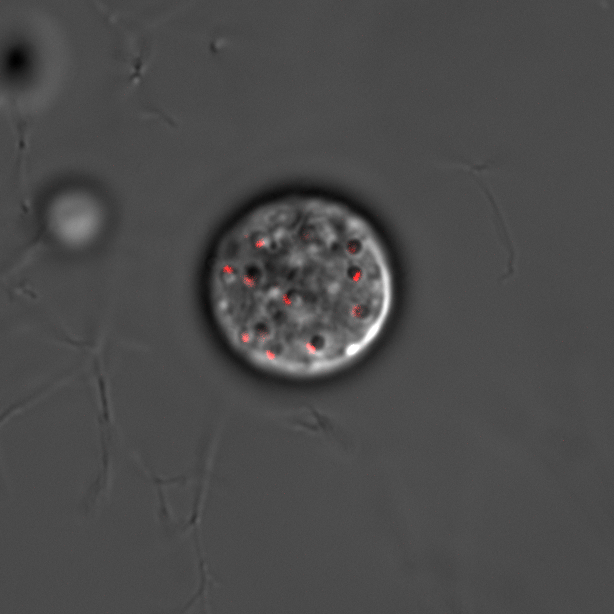

Supplement: Supplementary file 5 — Source data Fig. 3 [file 44318_2025_452_MOESM5_ESM.zip › 3E/Zstack28.png]

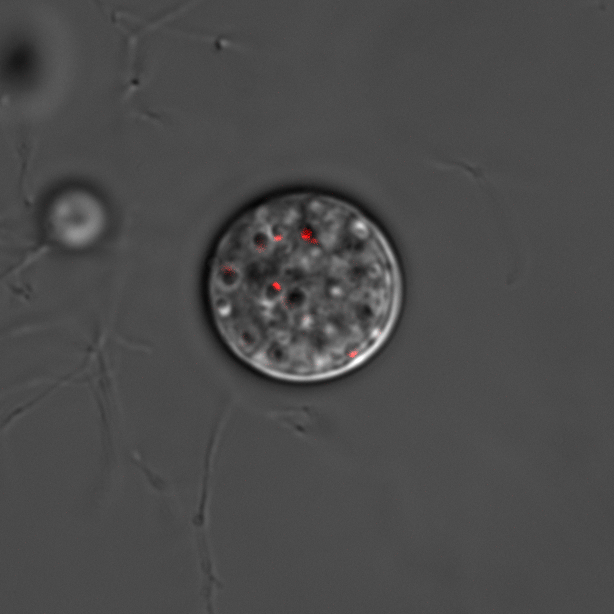

Supplement: Supplementary file 5 — Source data Fig. 3 [file 44318_2025_452_MOESM5_ESM.zip › 3E/Zstack42.png]

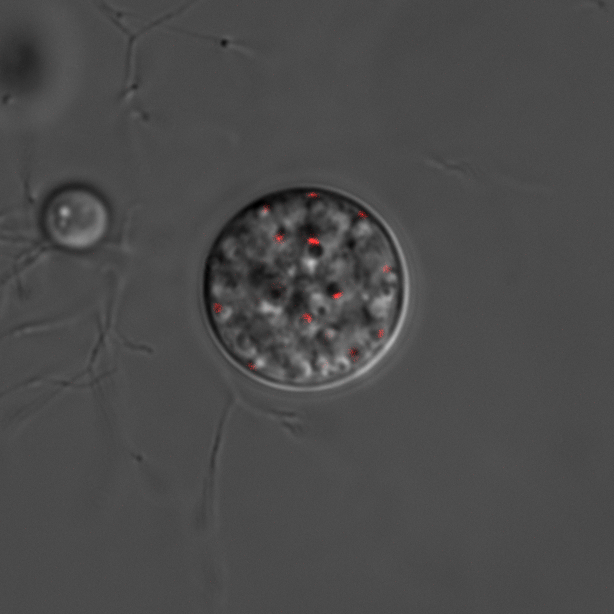

Supplement: Supplementary file 5 — Source data Fig. 3 [file 44318_2025_452_MOESM5_ESM.zip › 3E/Zstack56.png]

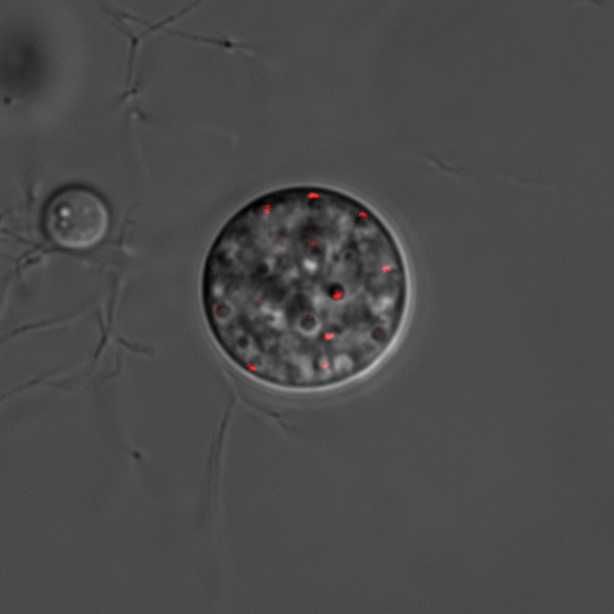

Supplement: Supplementary file 5 — Source data Fig. 3 [file 44318_2025_452_MOESM5_ESM.zip › 3E/Zstack63.png]

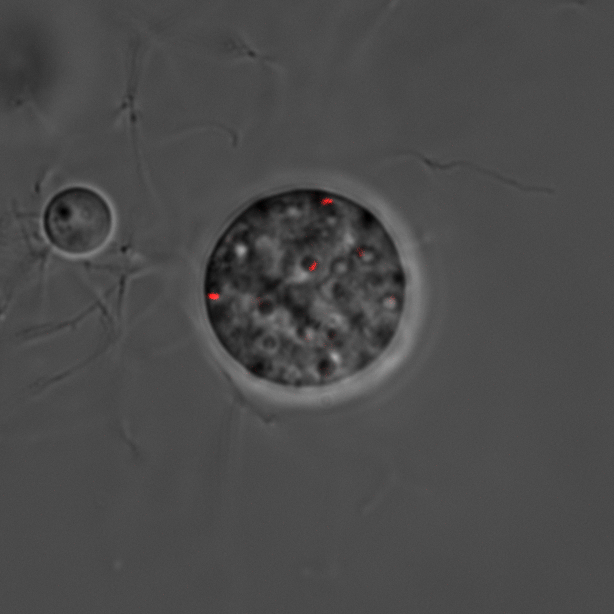

Supplement: Supplementary file 5 — Source data Fig. 3 [file 44318_2025_452_MOESM5_ESM.zip › 3E/Zstack84.png]

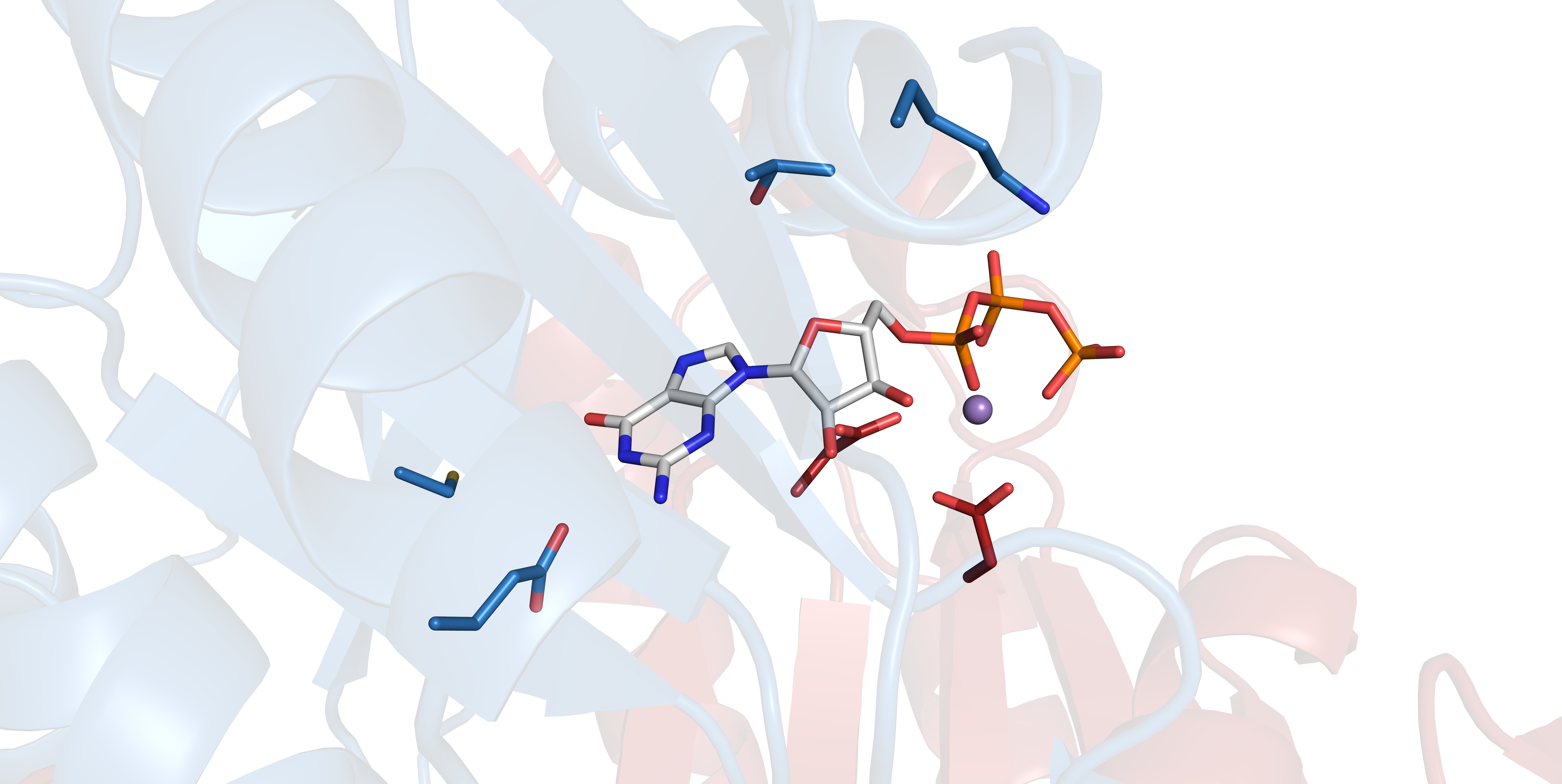

Supplement: Supplementary file 6 — Source data Fig. 4 [file 44318_2025_452_MOESM6_ESM.zip › 4A/NeoRBP_AF3.png]

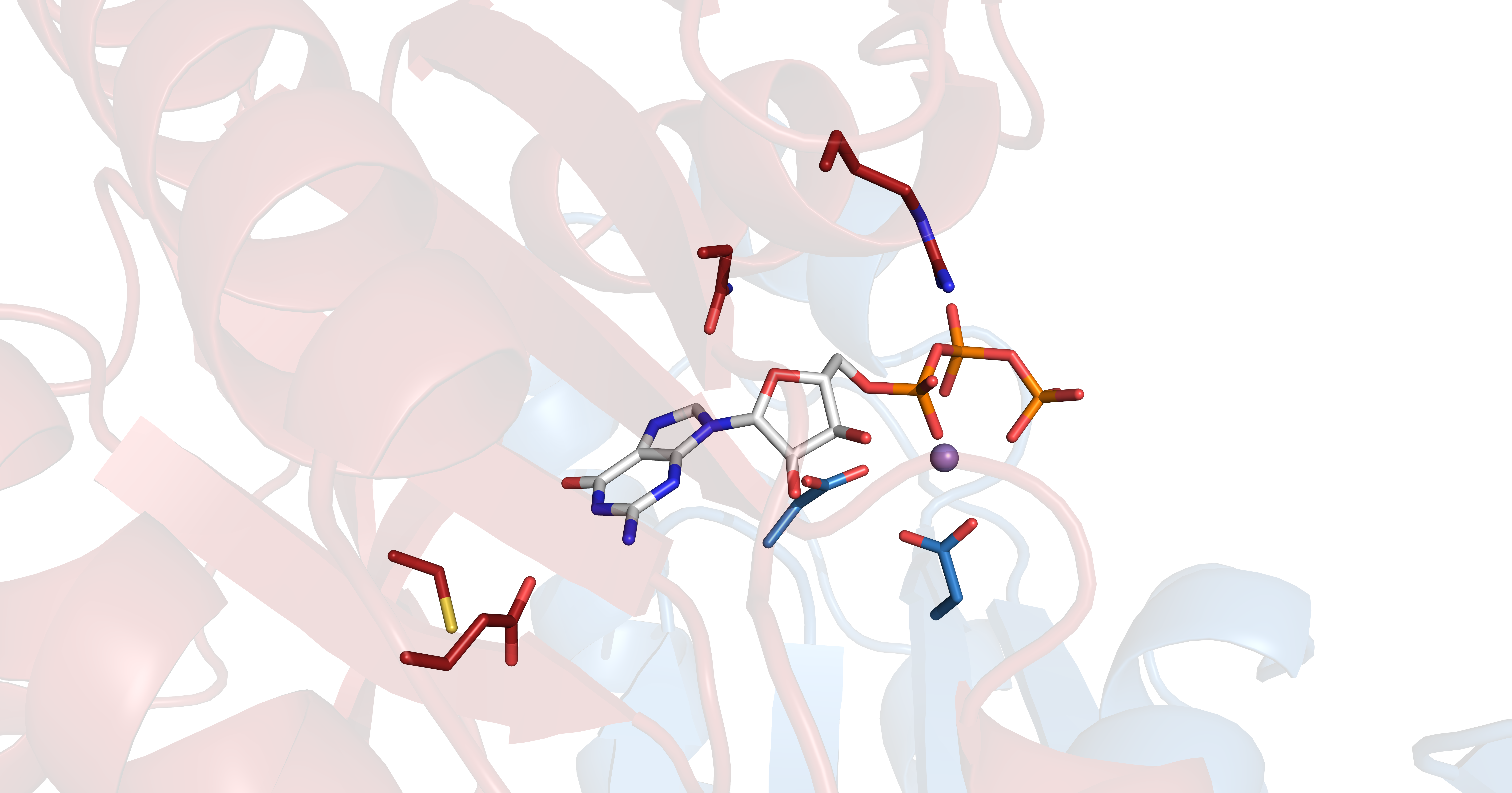

Supplement: Supplementary file 6 — Source data Fig. 4 [file 44318_2025_452_MOESM6_ESM.zip › 4A/RGC1BP_AF3.png]

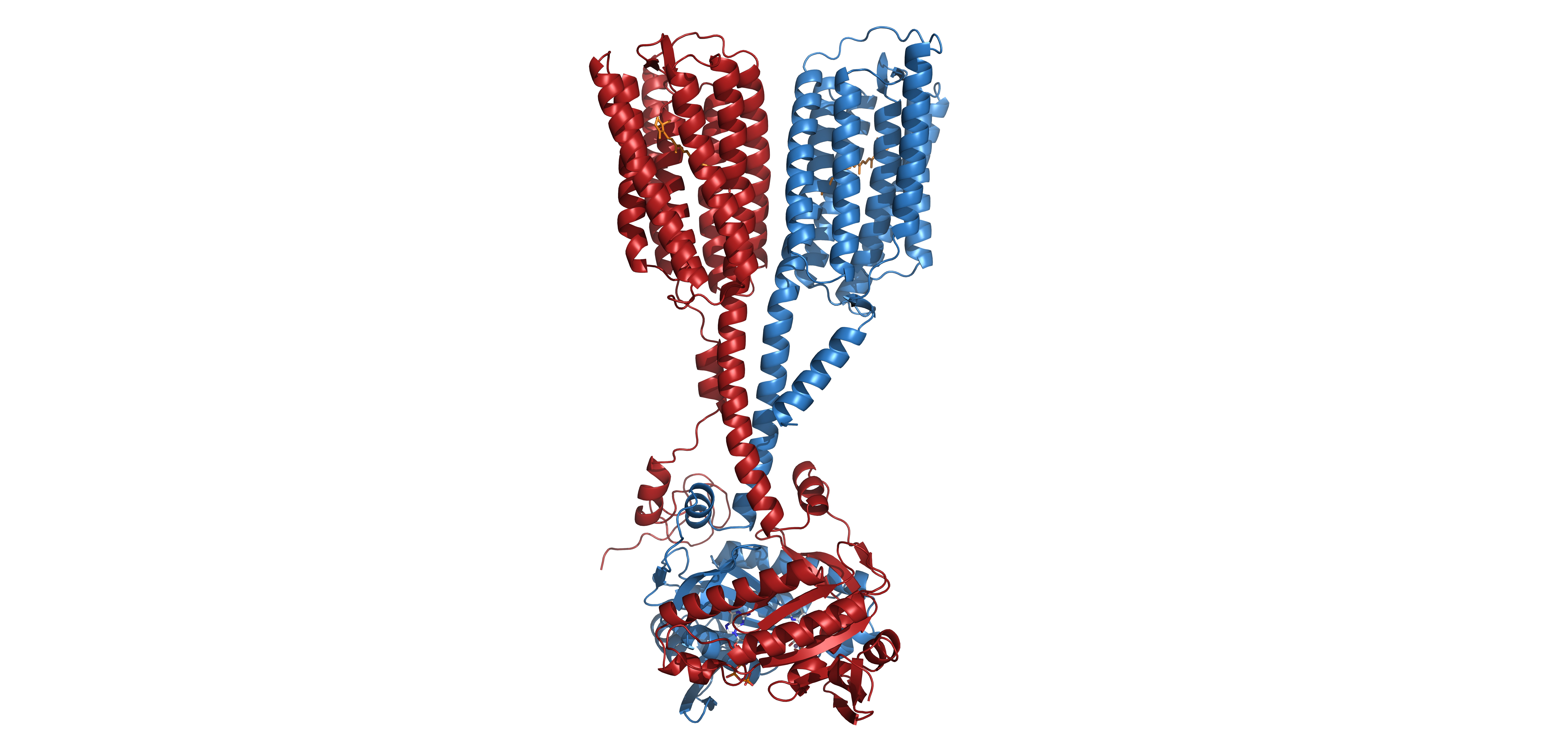

Supplement: Supplementary file 6 — Source data Fig. 4 [file 44318_2025_452_MOESM6_ESM.zip › 4A/RGC1NeoR-FL.png]

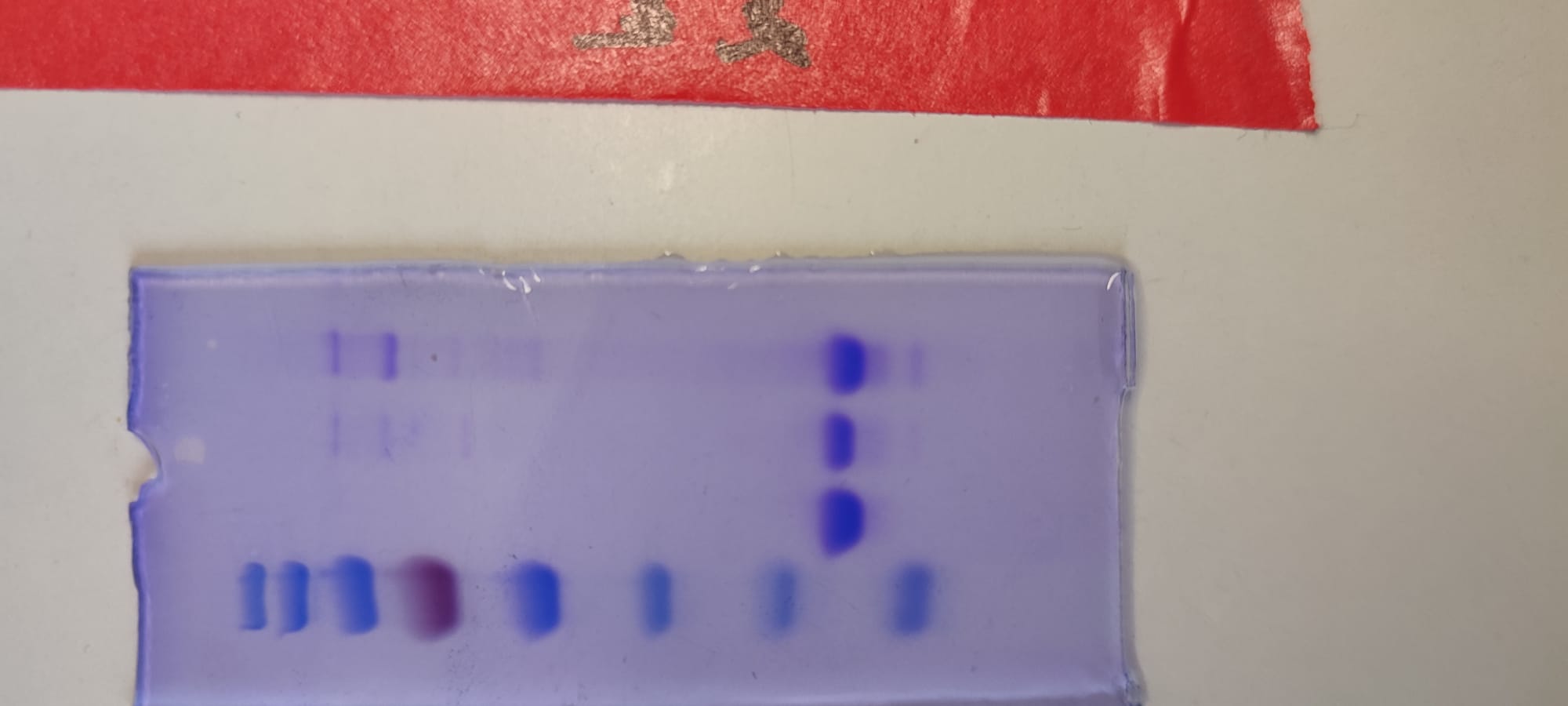

Supplement: Supplementary file 7 — Source data Fig. 5 [file 44318_2025_452_MOESM7_ESM.zip › 5A/Gel-image-Coomassie.jpg]

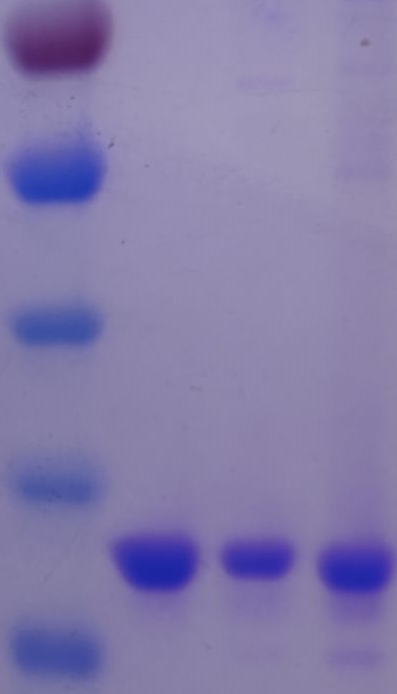

Supplement: Supplementary file 10 — Source Images [file 44318_2025_452_MOESM10_ESM.zip › Fig5A.png]
