# Supplementary material for: Super-resolution imaging of native fluorescent photoreceptors in chytrid fungal eyes
Source: EMBO J. 2025 May 27;44(14):4088–103. doi: 10.1038/s44318-025-00452-x (PMC12264004; doi:10.1038/s44318-025-00452-x)
Supplement: Supplementary file 11 — Expanded View Figures [file 44318_2025_452_MOESM11_ESM.pdf]

Expanded View Figures

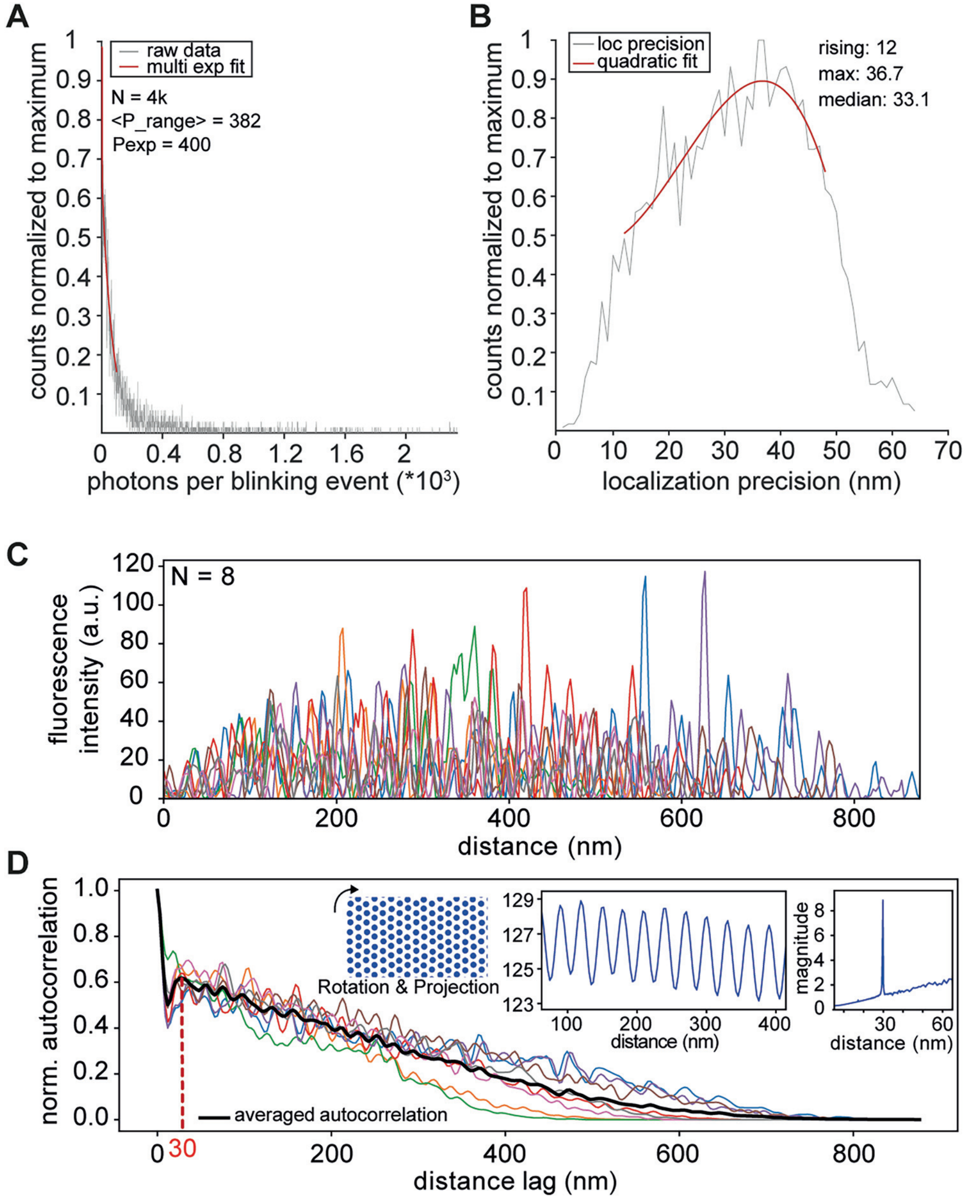

Figure EV1. Single-molecule localization microscopy data analysis, related to Fig. 3.

(**A**) Representative photon statistics for one individual zoospore. The SMAP software (Ries, 2020) was used to analyze blinking events recorded with the single-molecule localization microscope. The fits of the single blinking events resulted in x and y positions as well as background, photon number and localization precision. An exponential fit is applied to the distribution of photons per blinking event to determine the average photons per event. (**B**) Localization precision was calculated based on the photon count distribution, using the approach described in Ries (Ries, 2020) implemented in the SMAP software package. The localization precision was used to filter localizations with 50 nm and better, which serves as a threshold to exclude autofluorescence. The quadratic fit in (**B**) shows the estimated localization precision, with a median of 33.1 nm. (**C**) Background-corrected intensity profiles along the curved rumposome distributions highlight structured fluorescence patterns. (**D**) Autocorrelation functions from data in (**C**) are plotted over distance to assess periodicity in NeoR localizations, with the first peak detected at 30 nm (black line: averaged autocorrelation). The inset illustrates a simulated hexagonal distribution of NeoR localizations within the rumposome, based on experimentally determined spacing. The left panel shows the raw NeoR localization pattern, the middle panel presents the projection of the rumposome onto the x–y plane after rotation, and the right panel displays frequency analysis, confirming a periodic spatial pattern with a dominant peak at 30 nm. Localisation data were processed using the SMAP platform, ensuring robust drift correction and precision estimation.

 © The Author(s)

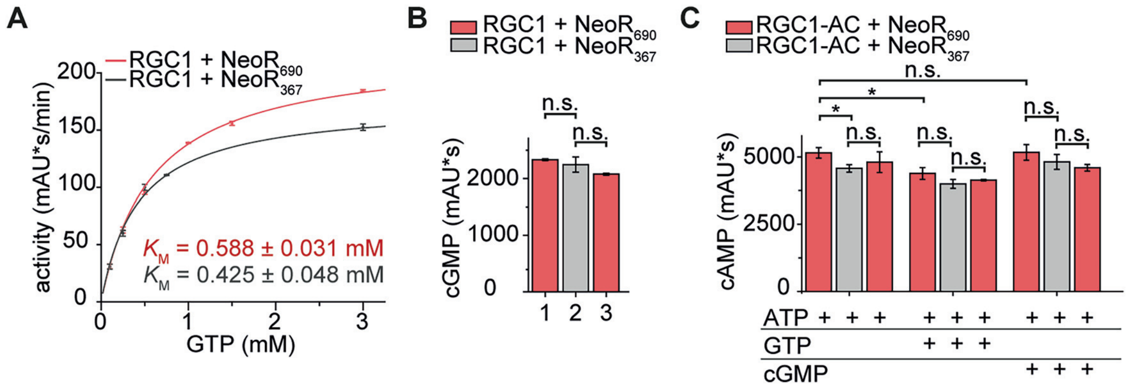

Figure EV2. Functional assays with photoswitched NeoR, related to Fig. 4.

(**A**) Michaelis-Menten kinetics of co-expressed RGC1/NeoR in HEK293T cells before and after photoconversion of NeoR, measured with 10 mM $MgCl_2$ and detected via reverse-phase HPLC. $n = 3$ (technical replicates), mean ± S.E. (**B**) End-point activity assay (light activation at 522 nm, 2 mM GTP/10 mM $MgCl_2$) with subsequent photoconversion of the NeoR subunit, 1: without pre-illumination, 2: photoconverted with 680 nm (20 min), 3: back-converted with 375 nm (1 min). $n = 3$ (technical replicates), mean ± S.D., unpaired t-test left-to-right: $p = 0.39$ (n.s. = not significant), $p = 0.12$ (n.s. = not significant). (**C**) End-point cyclase activity assay of co-expressed RGC1-AC/NeoR with 2 mM ATP/5 mM $MgCl_2$ and optionally cGMP or GTP (1 mM) added. NeoR was reversibly photoconverted using far-red (680 nm) and UV-light (375) as in (**B**). A minor decrease upon addition of GTP likely reflects competition with substrate ATP (Ruiz-Stewart et al, 2003). $n = 3$ (technical replicates), mean ± S.D., unpaired t-test left-to-right: $^*p = 0.01$, $^*p = 0.01$, $p > 0.05$ (n.s. = not significant, values are available in source data). Source data are available online for this figure.

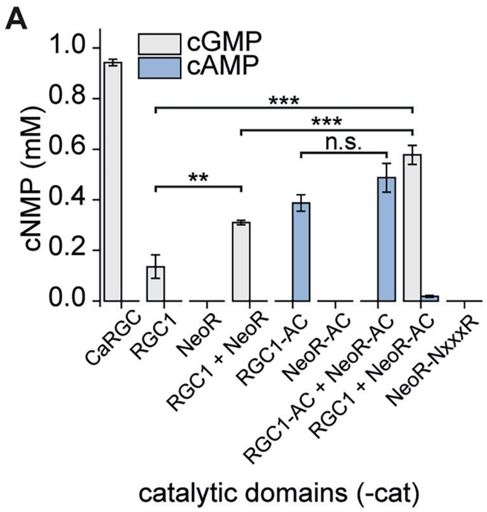

Figure EV3. Activity assays of soluble catalytic domain variants, related to Fig. 5.

(**A**) Endpoint enzyme assays (at 1 mM NTP/ 2 mM $MnCl_2$) with equimolar amounts of purified RGC-WT (*R. globosum*) catalytic domains and mutants, compared to homodimeric *Ca*RGC-cat (*C. anguillulae*). $n = 3$ (technical replicates), mean ± S.D., unpaired t-test left-to-right: $^{**}p = 3 \times 10^{-3}$, $^{***}p = 2 \times 10^{-4}$, $^{***}p = 3 \times 10^{-3}$, $p = 0.06$ (n.s. = not significant). Source data are available online for this figure.

 © The Author(s)
